# *EXTL2* and *EXTL3* inhibition with siRNAs as a promising substrate reduction therapy for Sanfilippo C syndrome

**DOI:** 10.1038/srep13654

**Published:** 2015-09-08

**Authors:** Isaac Canals, Noelia Benetó, Mónica Cozar, Lluïsa Vilageliu, Daniel Grinberg

**Affiliations:** 1 Departament de Genètica, Facultat de Biologia, Universitat de Barcelona, Barcelona, Spain; 2 Centro de Investigación Biomédica En Red de Enfermedades Raras (CIBERER), Spain; 3 Institut de Biomedicina de la Universitat de Barcelona (IBUB), Barcelona, Spain

## Abstract

Sanfilippo syndrome is a rare lysosomal storage disorder caused by an impaired degradation of heparan sulfate (HS). It presents severe and progressive neurodegeneration and currently there is no effective treatment. Substrate reduction therapy (SRT) may be a useful option for neurological disorders of this kind, and several approaches have been tested to date. Here we use different siRNAs targeting *EXTL2* and *EXTL3* genes, which are important for HS synthesis, as SRT in Sanfilippo C patients’ fibroblasts in order to decrease glycosaminoglycan (GAG) storage inside the lysosomes. The results show a high inhibition of the *EXTL* gene mRNAs (around 90%), a decrease in GAG synthesis after three days (30–60%) and a decrease in GAG storage after 14 days (up to 24%). Moreover, immunocytochemistry analyses showed a clear reversion of the phenotype after treatment. The *in vitro* inhibition of HS synthesis genes using siRNAs shown here is a first step in the development of a future therapeutic option for Sanfilippo C syndrome.

Sanfilippo C syndrome, or mucopolysaccharidosis (MPS) IIIC, is an autosomal recessive lysosomal storage disorder caused by mutations in the *HGSNAT* gene. This gene codes for an enzyme responsible for heparan sulfate (HS) degradation, and its dysfunction leads to the storage of partially degraded molecules inside the lysosomes^1^. HS is one of the most common glycosaminoglycans (GAGs) located in the extracellular matrix as a part of proteoglycans, which participate in many different cellular functions^2^. In the synthetic pathway of HS, there is an essential step in which the *EXTL* genes play a crucial role (reviewed in Ref. 3). In particular, EXTL2 and EXTL3 are key proteins involved in the HS chain elongation. Small interference RNAs (siRNAs), discovered in the late 1990s^4^, were shown to inhibit mammalian genes^5^, and may be applicable in substrate reduction therapy (SRT) for Sanfilippo C patients. For other types of MPS, two different approaches using siRNAs or shRNAs have been previously described which inhibit different genes in the HS synthetic pathway^6^,^7^.

In this study we used four different siRNAs, two of which inhibit *EXTL2* and the other two inhibit *EXTL3*. These siRNAs were transfected into fibroblasts of two patients in an attempt to reduce HS synthesis. All four siRNAs obtained a notable reduction in the mRNA levels of the corresponding gene and decreased the rates of GAG synthesis and storage.

## Materials and Methods

Fibroblasts from two patients were used in this study: patient SFC6 (genotype: c.633+1G > A/p.L445P) and patient SFC7 (genotype: c.372-2A > G/c.372-2A > G). The patients and the culture conditions were described in a previous work^8^. Fibroblasts were transfected with four different siRNAs using Lipofectamine 2000 as the transfection agent. Quantities of 5, 2.5 and 1.25 μl were used for 6-well, 12-well and 24-well plates respectively. Two Silencer® Select siRNAs (Ambion) for *EXTL2* (si4899 and si4900), two for *EXTL3* (si4901 and si4902) and one negative control (siC-) were used at a final concentration of 10 ηM. siRNA sequences are available on demand. Quantitative real-time PCR was performed using TaqMan® Gene Expression Assays for *EXTL2*, *EXTL3*, *HPRT* and *SDHA* genes, the last two as endogenous controls. PCRs were carried out in a LightCycler® 480 System (Roche). For every assay, the efficiency (E) of the reaction was calculated from a 7 points standard curve. The reaction efficiency was taken into account in the quantification cycle (Cq) calculation using LightCycler® 480 Software (release 1.5.0) (Roche). To confirm precision and reproducibility of real-time PCR the intra-assay precision was determined in 3 repeats within one LightCycler run. In the working conditions, the intra-assay coefficient of variation of all the assays at the working conditions was less than 1% and Cq standard deviation was smaller than 0.3.

The efficiency of GAG synthesis was quantified testing the cell incorporation of ^35^S sodium sulfate from the medium as previously described^6^. The sulfated GAG storage was quantified using the Rheumera® Proteoglycan Detection Kit (Astarte Biologics) following the manufacturer’s instructions. GAG extraction was performed as previously described^9^.

For heparan sulfate immunostainig, fibroblast cultures (untreated or treated as indicated above) were fixed in 4% PFA. Antibodies used were mouse anti-human HS (#H1890, 10E4 epitope, monoclonal antibody, USBiological) as a primary antibody, and donkey anti-mouse Cy2 (#715-225-150, polyclonal antibody, Jackson ImmunoResearch), as secondary antibody. For nucleus staining DAPI (Invitrogen) at 0.5 μg/ml was used. The slides were mounted with PVA:DABCO mounting medium. Images were acquired with a Leica DMIRB fluorescence microscope and analyzed with the Fiji software^10^.

All experimental protocols were approved by the Ethics Committee of the University of Barcelona and were conducted under the Declaration of Helsinki. Patients were encoded to protect their confidentiality, and informed consent obtained.

## Results

### Inhibition of *EXTL2* and *EXTL3* mRNA with specific siRNAs

All *EXTL* siRNAs were tested in fibroblasts from both patients and high inhibition levels were observed from day 3 to day 14 after transfection. All treated cells had expression levels of around 10% compared to cells treated with negative control siRNAs using *SDHA* as endongenous gene (Figure S1). Similar results were obtained with *HPRT* as endogenous gene (not shown).

### Decrease in GAG synthesis

**Figure 1:**
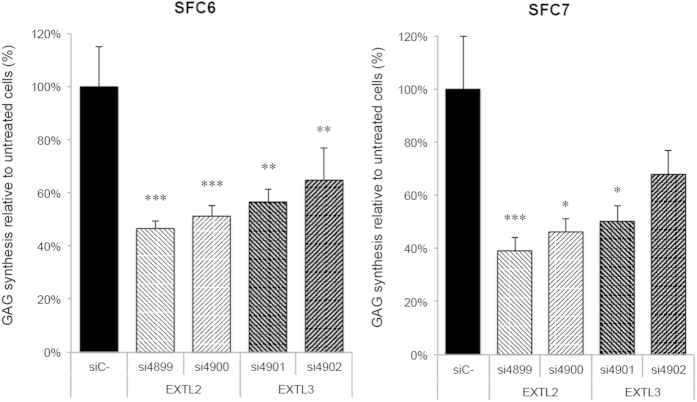
Inhibition of GAG synthesis. SFC6 and SFC7 fibroblasts were transfected with all four siRNAs and a negative control siRNA and after 3 days incorporation of ^35^S sodium sulfate was analysed. Results for both patients are the mean ± standard error of three experiments performed in quadruplicate and are expressed as disintegrations per minute per μg of DNA. Differences between *EXTL* siRNAs with respect to negative control siRNA were evaluated using the non-parametric Mann-Whitney U test, and statistical significance was set at p < 0.05 (*), p < 0.01 (**) or p < 0.001 (***).

After three days of transfection, fibroblasts showed a decrease in the incorporation of ^35^S sulfate of about 30% to 60% (depending on the siRNA used) compared to control samples (Fig. 1). Both patients showed similar results for each siRNA, indicating a more efficient inhibition of GAG synthesis for siRNAs designed to target the *EXTL2* gene (more than 50%) than in those targeting the *EXTL3* gene (less than 50%).

### Decrease in GAG storage

**Figure 2:**
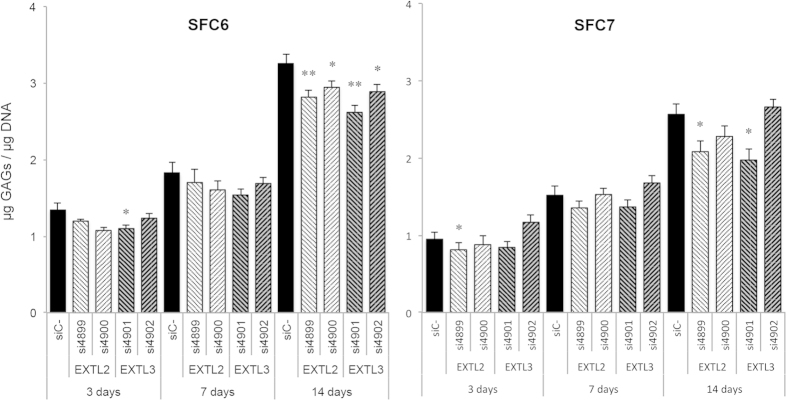
Decrease in GAG storage. SFC6 and SFC7 fibroblasts were transfected with all four siRNAs and a negative control siRNA and after 3, 7 and 14 days, GAG storage was analysed. Results for both patients are the mean ± standard error of three experiments performed in duplicate and are expressed in μg GAGs/μg DNA at 3 days, 7 days and 14 days after transfection. Differences between *EXTL* siRNAs respect to negative control siRNA were evaluated using the non-parametric Mann-Whitney U test, and statistical significance was set at p < 0.05 (*) or p < 0.01 (**).

Sulfated GAG storage was quantified in patients’ fibroblasts after treatment (Fig. 2). After three days, SFC6 fibroblasts showed 1.346 ± 0.085 μg GAGs/μg DNA, while treated fibroblasts presented between 1.075 and 1.235 μg GAGs/μg DNA. SFC7 fibroblasts had 0.953 ± 0.049 μg GAGs/μg DNA without treatment, and between 0.812 and 1.168 μg GAGs/μg DNA after treatment. After seven days, SFC6 fibroblasts showed 1.824 ± 0.136 μg GAGs/μg DNA, while treated fibroblasts presented between 1.538 and 1.701 μg GAGs/μg DNA. SFC7 fibroblasts had 1.521 ± 0.081 μg GAGs/μg DNA without treatment and between 1.354 and 1.677 μg GAGs/μg DNA after treatment. After 14 days, SFC6 fibroblasts showed 3.259 ± 0.085 μg GAGs/μg DNA, while treated fibroblasts presented between 2.618 and 2.945 μg GAGs/μg DNA. SFC7 fibroblasts had 2.569 ± 0.049 μg GAGs/μg DNA without treatment and between 1.973 and 2.66 μg GAGs/μg DNA after treatment. In general, patients’ storage decreased at each time point, reaching a maximum reduction of 24% for siRNA si4901 at 14 days in the fibroblasts of patient SFC7. GAG levels in WT fibroblasts were 0.796 ± 0.105 throughout the experiment.

**Figure 3:**
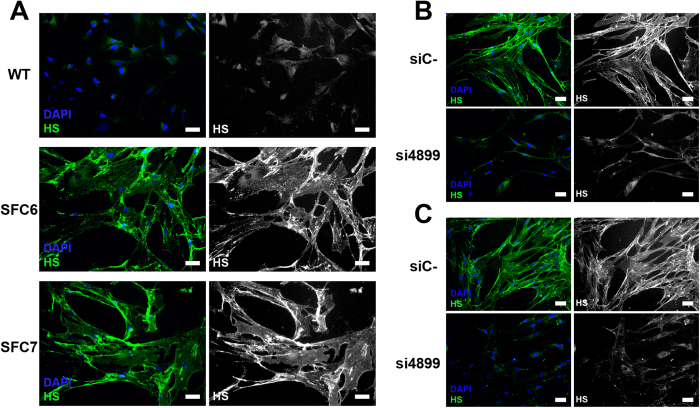
Heparan sulfate storage in WT and patients’ fibroblasts. (**A**) Immunocytochemistry analysis of HS accumulation (green) in untreated WT and SFC6 and SFC7 cells, using a specific anti-HS antibody. The same images are shown using the white channel to highlight HS staining. Fibroblasts from patient SFC6 (**B**) and patient SFC7 (**C**) were transfected with si4899 and with a negative control siRNA (siC-) for three days. HS was detected as in A, and shown in the green and white versions. Bar = 20 μm.

Heparan sulfate storage was evaluated through immunocytochemistry. Clear differences between WT and patients’ fibroblasts were observed after cells were fixed and stained with anti-HS antibodies (Fig. 3A). After a 3-day treatment with siRNA 4899 (as described above), patients’ fibroblasts showed a clear reduction in HS accumulation (Fig. 3B,C). siRNA 4899 was chosen for this experiment because it was the one that gave the best results in the reduction of GAG synthesis (see Fig. 1).

## Discussion

At present there is no effective treatment for Sanfilippo syndrome. In this report, different siRNAs were tested as SRT to inhibit key genes for HS synthesis in Sanfilippo C patients. A similar strategy has been applied previously, using shRNAs to inhibit *EXTL* genes^7^ and siRNAs to inhibit other genes participating in GAG synthesis^6^.

The siRNAs used in this study showed high mRNA inhibition capacity (around 90%) for as long as 14 days at low concentrations (10  nM). This is quite surprising, since we observed a relative loss of inhibition of the *GAPDH* gene (from 90% at day 3 to 60% at day 14, not shown). This long-lasting effect on the *EXTL* genes could be explained by their lower level of expression related to the *GAPDH* gene (less amount of siRNA would be enough to keep *EXTL* mRNA levels low). Besides, these new generation siRNAs are modified to achieve higher stability. Higher concentrations did not improve the result (data not shown), indicating the high efficiency of these siRNAs. These inhibition rates were higher than those previously obtained for the same genes with shRNAs^7^.

The results for GAG synthesis were interesting. After three days of transfection, all siRNAs achieved notable decreases in the synthetic pathway in both patients. Moreover, the reduction may have been even higher, considering that HS synthesis was inhibited but all types of GAG synthesis were quantified. Compared with previous results^6^,^7^, the higher mRNA inhibition levels obtained here achieved greater GAG synthesis inhibition. Another important point to note is that *EXTL2* inhibition seemed to be more efficient in decreasing GAG synthesis both in our study and in the study by Kaidonis *et al*.^7^, suggesting that *EXTL2* may be a better target candidate. It should be noted that at 7 and 14 days after transfection GAG synthesis was decreased in untreated cells, probably due to the fact that they reached confluence, which may have promoted a decrease in the synthetic pathway of extracellular matrix components such as GAGs. It would be interesting to be able to work with more suitable cell types – neural cells, for example - which are highly relevant to this disease, and have lower growth rates that would allow long-term studies.

Patients’ fibroblast cultures showed a pronounced increase in GAG storage with time, since these cells are not able to degrade HS. A slight decrease in storage has been detected after treatment with some of the siRNAs. The best results were observed at 14 days, although a trend towards a reduction was detected from three days of treatment onwards. We stress, again, that we decreased HS storage but quantified all GAG amounts, which may have led to an underestimation of the reduction. In this regard, when HS accumulation in the ECM was evaluated by immunocytochemistry, using HS-specific antibodies, a clear reduction of the accumulation was observed after three days of treatment.

Taken together, our results indicate that these siRNAs promote a reduction in mRNA levels of target genes, a notable reduction in the GAG synthetic pathway after 3 days, specifically of HS, as shown by immunocytochemistry, and a slower accumulation rate in patients’ cells over two weeks. Although further research is needed, RNAi-based therapies is a promising approach, probably to be used as a complementary therapy to obtain synergic effects with other treatments in order to accelerate the rate of HS degradation and/or excretion out of the cell. The search for novel shRNAs, stable and highly inhibitory for *EXTL2* (the best target, according to our results and those of other authors) is a necessary next step on the way to achieving a successful SRT for Sanfilippo syndrome.

## Additional Information

**How to cite this article**: Canals, I. *et al*. *EXTL2* and *EXTL3* inhibition with siRNAs as a promising substrate reduction therapy for Sanfilippo C syndrome. *Sci. Rep*. **5**, 13654; doi: 10.1038/srep13654 (2015).

## Supplementary Material

Supplementary Information
