# Supplementary material for: EXTL2 and EXTL3 inhibition with siRNAs as a promising substrate reduction therapy for Sanfilippo C syndrome
Source: Sci Rep. 2015 Sep 8;5:13654. doi: 10.1038/srep13654 (PMC4561882; doi:10.1038/srep13654)
Supplement: Supplementary Information [file srep13654-s1.pdf]

EXTL2 and *EXTL3* inhibition with siRNAs as a promising substrate reduction therapy for Sanfilippo C syndrome.

Isaac Canals[1,2,3], Noelia Benetó[1,2,3], Mónica Cozar[1,2,3,], Lluïsa Vilageliu[1,2,3*], Daniel Grinberg[1,2,3*#].

[1] Departament de Genètica, Facultat de Biologia, Universitat de Barcelona, Barcelona, Spain
[2] Centro de Investigación Biomédica En Red de Enfermedades Raras (CIBERER), Spain
[3] Institut de Biomedicina de la Universitat de Barcelona (IBUB), Barcelona, Spain
* Co-last authors.
[#] Corresponding author (dgrinberg@ub.edu)

Supplementary material

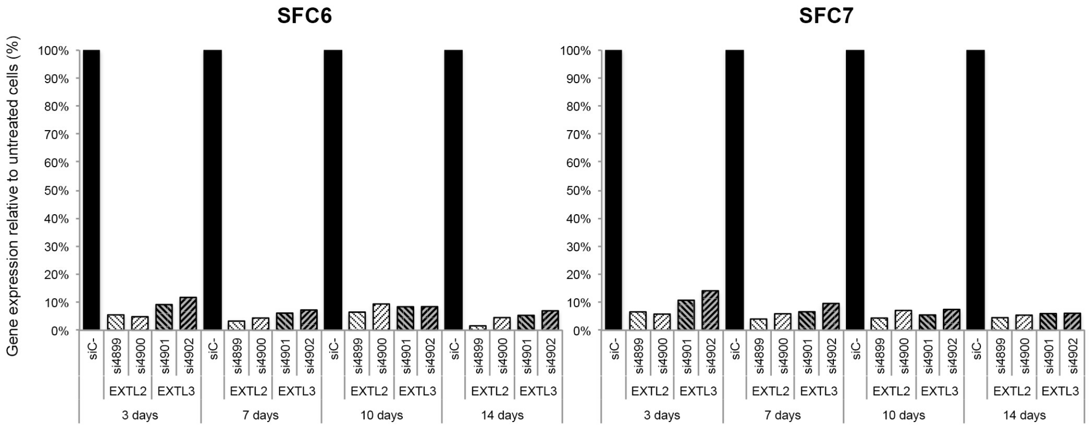

Figure S1 *EXTL2* and *EXTL3* gene silencing. SFC6 and SFC7 fibroblasts were transfected with all four siRNAs and a negative control siRNA, and after 3, 7, 10 and 14 days, gene expression was analysed by real-time PCR. Results are expressed as the percentage of gene expression compared to cells transfected with the negative control siRNA and using two different reference genes (*HPRT* and *SDHA*).
